# Inflammatory factor receptor Toll‐like receptor 4 controls telomeres through heterochromatin protein 1 isoforms in liver cancer stem cell

**DOI:** 10.1111/jcmm.13606

**Published:** 2018-03-30

**Authors:** Qidi Zheng, Jie Xu, Zhuojia Lin, Yanan Lu, Xiaoru Xin, Xiaonan Li, Yuxin Yang, Qiuyu Meng, Chen Wang, Wujun Xiong, Dongdong Lu

**Affiliations:** ^1^ Research Center for Translational Medicine at Shanghai East Hospital School of Life Science and Technology Tongji University Shanghai China; ^2^ Department of Hepatology Shanghai East Hospital Tongji University School of Medicine Shanghai China

**Keywords:** cancer stem cells, heterochromatin protein 1, telomeres, Toll‐like receptor 4

## Abstract

Toll‐like receptor 4 (TLR4) which acts as a receptor for lipopolysaccharide (LPS) has been reported to be involved in carcinogenesis. However, the regulatory mechanism of it has not been elucidated. Herein, we demonstrate that TLR4 promotes the malignant growth of liver cancer stem cells. Mechanistically, TLR4 promotes the expression of histone‐lysine N‐methyltransferase (SUV39 h2) and increases the formation of trimethyl histone H3 lysine 9‐heterochromatin protein 1‐telomere repeat binding factor 2 (H3K9me3‐HP1‐TRF2) complex at the telomeric locus under mediation by long non coding RNA urothelial cancer‐associated 1 (CUDR). At the telomeric locus, this complex promotes binding of POT1, pPOT1, Exo1, pExo1, SNM1B and pSNM1B but prevents binding of CST/AAF to telomere, thus controlling telomere and maintaining telomere length. Furthermore, TLR4 enhances interaction between HP1α and DNA methyltransferase (DNMT3b), which limits RNA polymerase II deposition on the telomeric repeat‐containing RNA (TERRA) promoter region and its elongation, thus inhibiting transcription of TERRA. Ultimately, TLR4 enhances the telomerase activity by reducing the interplay between telomerase reverse transcriptase catalytic subunit (TERT) and TERRA. More importantly, our results reveal that tri‐complexes of HP1 isoforms (α, β and γ) are required for the oncogenic action of TLR4. This study elucidates a novel protection mechanism of TLR4 in liver cancer stem cells and suggests that TLR4 can be used as a novel therapeutic target for liver cancer.

## 1 INTRODUCTION

Toll‐like receptor (TLR) family plays a fundamental role in pathogen recognition and activation of innate immunity. Toll‐like receptor 4 (TLR4) is often overexpressed in malignant and tumour‐infiltrating immune cells. Of particular importance is TLR4‐mediated recruitment of endothelial progenitors derived from immature myeloid cells^1^; especially, TLR4 plays a crucial role in mesenchymal stem cell (MSC)‐induced inhibition of natural killer (NK) cell function.^2^ Moreover, excessive TLR4 expression is accompanied by chromatin decompaction and demethylation of the proximal TLR4 promoter.^3^ In addition, TLR4 signalling via NANOG cooperates with Signal Transducers and Activators of Transcription 3 (STAT3) to promote formation of tumour‐initiating stem‐like cells in livers.^4^ It also suggests that TLR4 drives breast cancer cell growth differentially depending on the presence of tumour suppressor P53.^5^

Three members of the human heterochromatin protein 1 (HP1) family (HP1α, HP1β and HP1γ) are involved in chromatin packing and epigenetic gene regulation.^6^ Emerging evidence has shown that HP1α plays a unique biological role in breast cancer‐related processes and particularly in epigenetic control mechanisms involved in aberrant cell proliferation and metastasis.^7^ α, β and γ proteins of HP1 family selectively bind to methylated lysine 9 of histone H3 via their chromo‐domains. Also, HP1γ recognition of lysine 9 in the histone H3 tail in different nucleosome structures plays a role in reading the histone code.^8^ Notably, both HP1α and Argonaute 1 (AGO1) are involved in chromatin‐related splicing regulation.^9^ Moreover, HP1 regulates alternative splicing in a methylation‐dependent manner by recruiting splicing factors to its methylated form.^10^

Telomere length and telomerase activity normalize after several rounds of passaging, which is consistent with the ability of Pim‐1 (PIM kinases, a family of Ser/Thr kinases) to transiently increase mitosis.^11^ Telomere repeat binding factors 1 (TRF1) and 2 (TRF2) binding to telomeres are modulated by nucleosomal organization.^12^ The stability of mammalian telomeres depends on TRF2, which prevents inappropriate repair and checkpoint activation.^13^ Upon telomere shortening or telomere uncapping induced by loss of TRF2, telomeres elicit a DNA damage response leading to cellular senescence.^14^ The human telomerase RNA component (hTR) activates the DNA‐dependent protein kinase to phosphorylate heterogeneous nuclear ribonucleoprotein.^15^ In particular, long non coding RNA urothelial cancer‐associated 1 (lncRNA CUDR) promotes liver cancer stem cell growth through up‐regulating telomerase reverse transcriptase catalytic subunit (TERT) and C‐Myc.^16^

Long non coding telomeric repeat‐containing RNA (TERRA) is involved in telomere maintenance in a telomerase‐dependent and a telomerase‐independent manner during replicative senescence and cancer.^17^ TERRA participates in the regulation of telomere length, telomerase activity and heterochromatinization.^18^ Some research shows that telomeres are protected from hyper‐resection through the repression of the Ataxia‐telangiectasia‐mutated (ATM) and ATR kinases by TRF2 and tripeptidyl peptidase 1‐bound telomeric DNA binding proteins 1a/b (TPP1‐bound POT1a/b), respectively.^19^ Moreover, Exo1 extensively resects both telomere ends, generating transient long 3′ overhangs in S phase/G2 phase checkpoint. CST/AAF, a DNA polα primase accessory factor, binds POT1b and shortens the extended overhangs produced by Exo1, likely through fill‐in synthesis.^20^, ^21^ Furthermore, genetic variants in telomere maintenance genes are associated with genomic instability, cancer risk and cancer metastasis.^22^, ^23^, ^24^

In this study, we attempted to elucidate TLR4 functions during the malignant growth of liver cancer stem cells. Specifically, we investigated whether TLR4 promotes the malignant proliferation and growth of liver cancer stem cells in vitro and in vivo*,* and investigated its potential role in the malignant transformation of liver stem cells by analysing the cascade of TLR4‐HP1 (α, β and γ)‐telomere signalling.

## 2 MATERIALS AND METHODS

### 2.1 Human liver cancer stem cell (hLCSC) line sorting

CD133/CD44/CD24/EpCAM MicroBead Kits (MACS^®^ Technology) were purchased from Miltenyi Biotec Inc. (Boston, USA). Human liver cancer cell line Huh7 cell suspension was centrifuged at 300 *g* for 10 minutes, and cell pellet was resuspended in 300 μL buffer (for total 10^8^ cells). Then, 100 μL FcR Blocking Reagent (for total 10^8^ cells) and 100 μL CD133/CD44/CD24/EpCAM MicroBeads (for total 10^8^ cells) were added into the buffer. The solution was mixed well and incubated for 30 minutes in the refrigerator (2‐8°C). After that, cells were washed by adding 1‐2 mL buffer (total 10^8^ cells) and centrifuged at 300 *g* for 10 minutes. Then, these cells were resuspended in 500 μL buffer. An appropriate MACS Column and MACS Separator were chosen according to the amount of total cells and the amount of CD133^+^/CD44^+^/CD24^+^/EpCAM^+^ cells.

### 2.2 Cell lines and plasmids

hLCSC were maintained in Dulbecco's modified Eagle's medium (Gibco BRL Life Technologies) supplemented with 10% heat‐inactivated foetal bovine serum (Gibco BRL Life Technologies) in a humidified atmosphere of 5% CO_2_ at 37°C. pCMV6‐AC‐GFP and pGFP‐V‐RS were purchased from OriGene (Rockville, MD, USA). pcDNA3.1‐DNMT3b, pcDNA3.1‐HP1α, pcDNA3.1‐HP1β, pcDNA3.1‐HP1γ were purchased from Addgene (Cambridge MA, USA). pCMV6‐AC‐GFP‐TLR4, pGFP‐V‐RS‐TLR4, pGFP‐V‐RS‐HP1α, pGFP‐V‐RS‐HP1β, pGFP‐V‐RS‐HP1γ were prepared in our laboratory.

### 2.3 Co‐immunoprecipitation (IP)

Co‐immunoprecipitation was carried out according to methodology as previously described.^25^ Briefly, cell lysates were incubated with 2 μg antibody or normal mouse/rabbit IgG under rotation for 4 hours at 4°C. Then, the immunoprecipitates were incubated with 30 μL protein G/A‐plus agarose beads under rotation overnight at 4°C. The precipitates were washed with beads solution for five times, and then, the precipitates were resuspended in 60 μL 2 × SDS‐PAGE sample loading buffer. Western blotting was then performed.

### 2.4 Chromatin immunoprecipitation (ChIP) assay

Chromatin immunoprecipitation was carried out according to methodology as previously described.^26^ Briefly, cells were cross‐linked with 1% (v/v) formaldehyde (Sigma‐Aldrich) for 10 minutes at room temperature. Chromatin extracts were immunoprecipitated with specific antibody on protein‐A/G‐sepharose beads. After washing and de‐cross‐linking, the ChIP DNA was detected by PCR.

### 2.5 Quantitative telomerase detection

Telomerase activity was measured by Quantitative Telomerase Detection Kit (MT3010) according to manufacturer's instructions (US Biomax, Inc.).

### 2.6 Telomere length assay

Telomere length assay using Telo TAGGG PCR ELISA plus kit was performed according to manufacturer's instructions (Roche). A standard curve was established by dilution of known quantities of a synthesized 84‐mer oligonucleotide containing only TTAGGG repeats.

### 2.7 Methylation analysis

Methylated DNA Immunoprecipitation (MeDIP)‐Dot blot‐Western blotting was performed with anti‐5‐methylcytosine (5‐mC) and methylation analysis by MspI plus BamHI digestion.

### 2.8 Xenograft transplantation in vivo

Four‐week‐old athymic BALB/c mice (24 mice) were injected subcutaneously with hLCSCs in the armpit area. The mice were observed over 4 weeks and then killed for the purpose of recovering the tumours. The wet weight of each tumour was determined for each mouse. A portion of each tumour was fixed in 4% paraformaldehyde and embedded in paraffin for histological haematoxylin‐eosin (HE) staining. The use of mice in this work was reviewed and approved by the Institutional Animal Care and Use Committee in accordance with guidelines of China National Institutes of Health.

## 3 RESULTS

### 3.1 TLR4 promotes malignant proliferation of hLCSCs in vitro

**Figure 1:**
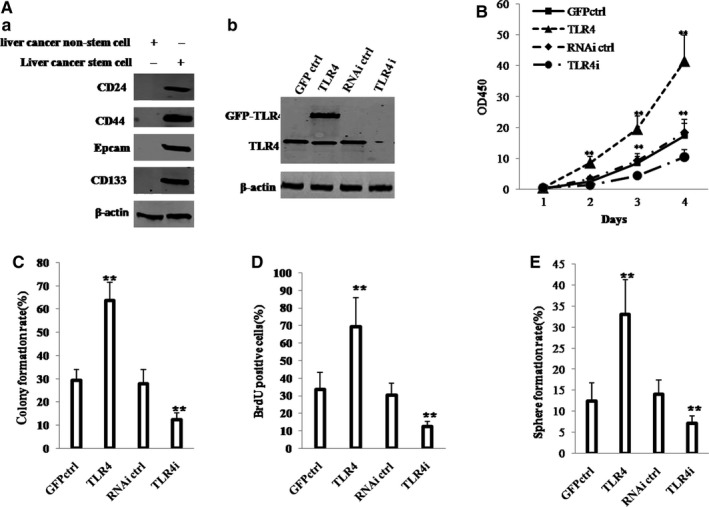
TLR4 accelerates hLCSCs growth in vitro. A, (a) Western blotting analysis of expression of CD133, CD44, CD24 and EpCAM in hLCSCs and non‐hLCSCs. (b) Western blotting analysis of TLR4 expression in four hLCSC lines. β‐Actin was used as internal control. B, Cell growth assay using CCK8. C, Soft‐agar colony formation assay. D, S phase cells assay using BrdU. E, Cell sphere formation ability. Each value was presented as mean ± standard error of the mean (SEM). mean ± SEM. **P < .01; *P < .05. For all Western blotting, we repeated the experiments for three times. We measured grey value of the bands for quantification. Each value was presented as mean ± standard error of the mean (SEM) (Student's *t* test)

We used CD133/CD44/CD24/EpCAM MicroBeads to isolate hLCSCs from human liver cancer cell line Huh7 by detecting the markers of hLCSCs, including CD133, CD44, CD24 and EpCAM. As shown in Figure 1Aa, CD133, CD44, CD24 and EpCAM were expressed in hLCSCs. However, these were not expressed in non‐hLCSCs. We detected the expression of TLR4, MD2 and CD14 in non‐transfected hLCSCs and non‐hLCSCs by Western blotting. The results showed that the expression of TLR4 in hLCSCs was significantly higher than that in non‐hLCSCs, and the expression of MD2 or CD14 in hLCSCs was lower than that in non‐hLCSCs (Figure S1).

Then, we established four stable hLCSC lines transfected with pCMV6‐AC‐GFP (GFP ctrl group), pCMV6‐AC‐GFP‐TLR4 (TLR4 group), pGFP‐V‐RS (RNAi ctrl group) and pGFP‐V‐RS‐TLR4 (TLR4i group), respectively. As shown in Figure 1Ab, compared with GFP ctrl group, TLR4 expression was significantly enhanced in TLR4 group. However, TLR4 expression was significantly reduced in TLR4i group compared with RNAi ctrl group. Furthermore, the expression of TLR4 on cell surface was significantly increased in TLR4 overexpressing hLCSC and was significantly decreased in TLR4 knocked‐down hLCSC compared to control (Figure S2).

As shown in Figure 1B, compared with growth of hLCSCs in GFP ctrl group (*P* < .01), that in TLR4 group was significantly increased; however, compared with growth of hLCSCs in RNAi ctrl group, that in TLR4i group was significantly decreased. In addition, compared with soft‐agar colony formation of hLCSCs in GFP ctrl group (*P* < .01), that in TLR4 group was significantly increased; however, compared with soft‐agar colony formation of hLCSCs in RNAi ctrl group (*P* < .01), that in TLR4i group was significantly decreased (Figure 1C). Furthermore, compared with the proportion of BrdU‐positive cells in GFP ctrl group, that in TLR4 group was significantly increased; however, compared with the proportion of BrdU‐positive cells in RNAi ctrl group (*P* < .01), that in TLR4i group was significantly decreased (Figure 1D). Strikingly, sphere formation rate of hLCSCs was significantly higher in TLR4 group than in the GFP ctrl group (*P* < .01), whereas that was lower in TLR4i group than in the RNAi ctrl group (*P* < .01) (Figure 1E). Moreover, our results showed that excessive TLR4 significantly increased the interaction between TLR4 and MD2 (a TLR4 ligand) or TLR4 and MyD88 (a TLR4 dimer ligand) (Figure S3A). Excessive TLR4 significantly promoted the colony formation ability of liver cancer stem cell (37.79 ± 3.29 vs 70.66 ± 7.53%, *P* = .0094 < .01). However, when MD2 or MyD88 was knocked down in the TLR4 overexpressing liver cancer stem cells, excessive TLR4 could significantly not alter the growth and the colony formation ability of liver cancer stem cell (TLR4 + MD2i:37.79 ± 3.29 vs 33.76 ± 5.333%, *P* = .243 > .05; TLR4 + MyD88i: 37.79 ± 3.29 vs 35.2 ± 8.67%, *P* = .362 > .05) (Figure S3B). These results suggest that TLR4 promotes the proliferation of liver cancer stem cells in vitro.

### 3.2 TLR4 accelerates growth of hLCSCs in vivo

**Figure 2:**
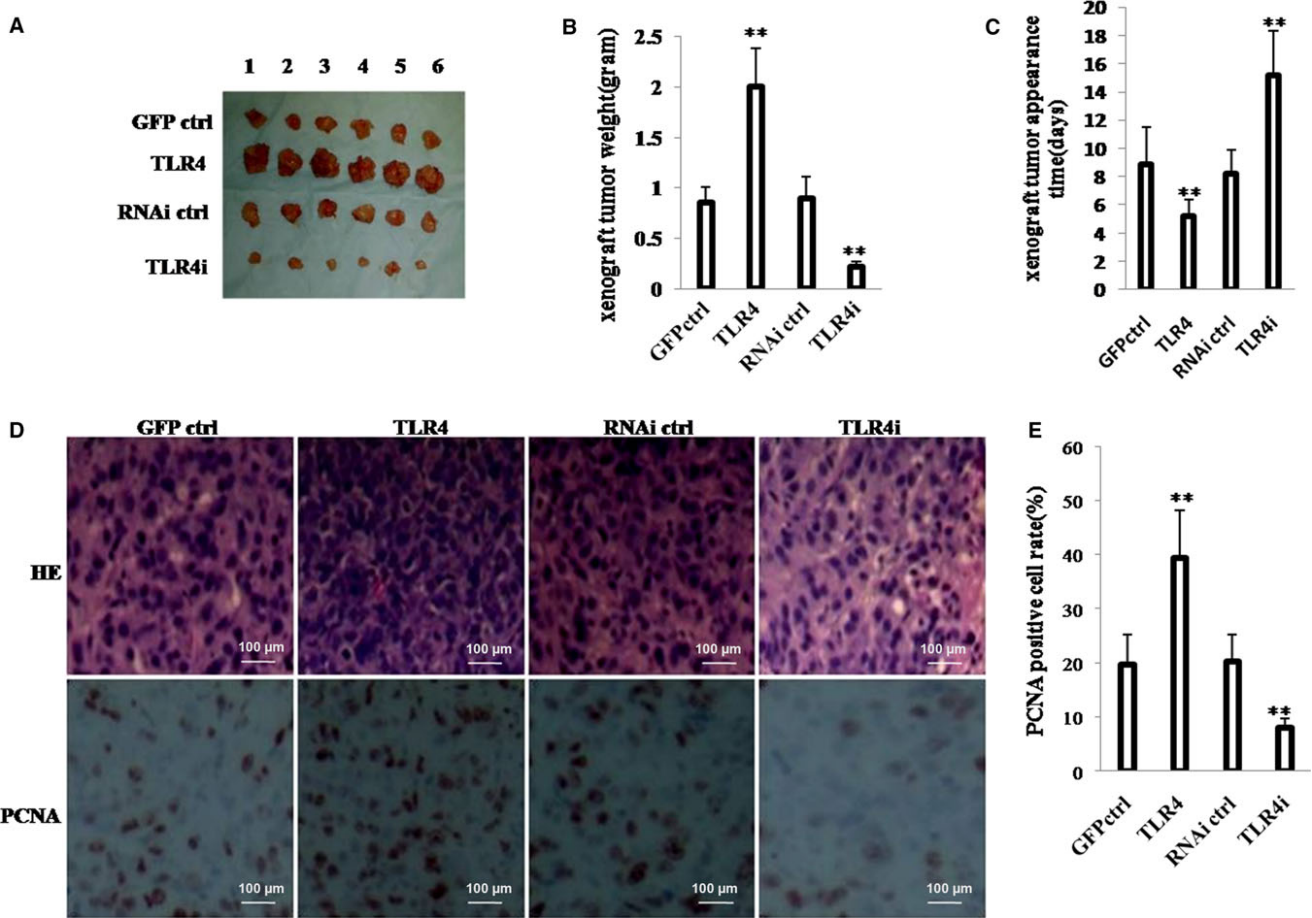
TLR4 accelerates hLCSCs growth in vivo. A, The photograph of xenograft tumours derived from four hLCSC lines injected into mice. B, The wet weight of xenograft tumours. C, The appearance time of xenograft tumours. D, Histological haematoxylin‐eosin (HE) staining (upper pictures) and anti‐PCNA immunostaining (lower pictures) of xenograft tumours. (original magnification × 100). E, PCNA‐positive cell analysis of xenograft tumours. Each value was presented as mean ± standard error of the mean (SEM). mean ± SEM. **P < .01; *P < .05. For all Western blotting, we repeated the experiments for three times. We measured grey value of the bands for quantification. Each value was presented as mean ± standard error of the mean (SEM) (Student's *t* test).

To further explore the effect of TLR4 on hLCSCs in vivo, the four stable hLCSCs lines were injected subcutaneously into athymic BALB/c mice, respectively. As shown in Figure 2A,B, compared with the weight of xenograft tumour in GFP ctrl group (*P* < .01), that in TLR4 group was increased approximately by 2.5‐fold; however, compared with the weight of xenograft tumour in RNAi ctrl group (*P* < .01), that in TLR4i group was decreased approximately by three‐fourths. In addition, the xenograft tumours appeared earlier in TLR4 group than in GFP ctrl group (*P* < .05), whereas those appeared later in TLR4i group than in RNAi ctrl group (*P* < .01) (Figure 2C). Furthermore, xenograft tumour differentiation was poorer in TLR4 group than in GFP ctrl group, whereas that was better in TLR4i group than in RNAi ctrl group (Figure 2D, upper pictures). Strikingly, the percentage of proliferating cell nuclear antigen (PCNA)‐positive cells from xenograft tumours was significantly higher in TLR4 group than in GFP ctrl group (*P* < .01), whereas that was significantly lower in TLR4i group than in RNAi ctrl group (*P* < .01) (Figure 2D, lower pictures; Figure 2E). These results demonstrate that TLR4 accelerates malignant growth of liver cancer stem cells in vivo.

### 3.3 TLR4 enhances the interplay between HP1 isoforms and H3K9me3

**Figure 3:**
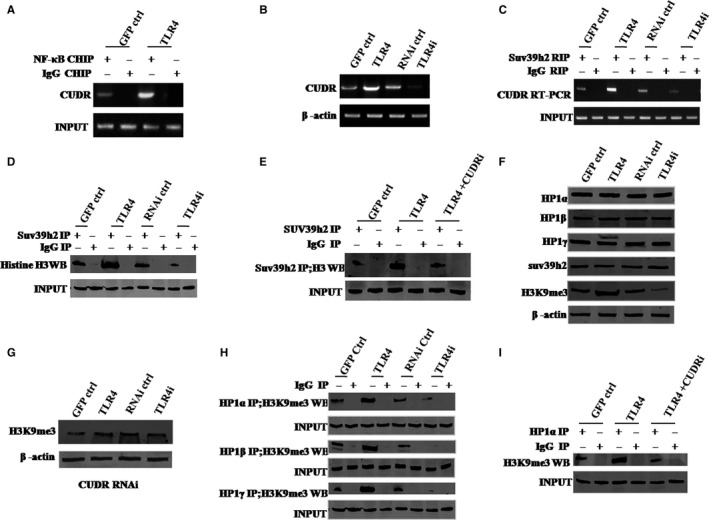
TLR4 increases interplay between HP1 isoforms and H3K9me3 via CUDR. A, Chromatin Immunoprecipitation (ChIP) with anti‐NF‐κB followed by PCR with CUDR promoter primers. IgG ChIP served as negative control. B, RT‐PCR analysis of CUDR mRNA. β‐Actin served as internal control. C, RNA Immunoprecipitation (RIP) with anti‐SUV39 h1 followed by RT‐PCR with CUDR promoter primers. IgG RIP served as the negative control. D, Co‐immunoprecipitation (Co‐IP) with anti‐SUV39 h2 followed by Western blotting with antihistone. IgG IP served as the negative control. Western blotting with anti‐SUV39 h2 served as INPUT. E, Co‐IP with anti‐SUV39 h2 followed by Western blotting with antihistone H3. IgG IP served as negative control. Western blotting with anti‐SUV39 h2 served as INPUT. F, Western blotting with anti‐HP1α, anti‐HP1β, anti‐HP1γ, anti‐H3K9me3, anti‐SUV39 h2. β‐Actin served as an internal control. G, Western blotting with anti‐H3K9me3 (four hLCSC lines with CUDR being depleted). β‐Actin was the internal control. H, Co‐IP with anti‐H3K9me3 followed by Western blotting with anti‐HP1α, anti‐HP1β and anti‐HP1γ. IgG IP served as negative control. Western blotting with anti‐HP1α, anti‐HP1β, anti‐HP1γ served as INPUT. I, Co‐IP with anti‐H3K9me3 followed by Western blotting with anti‐HP1α. IgG IP was used as negative control. Western blotting with anti‐HP1α as INPUT. Each value was presented as mean ± standard error of the mean (SEM). Mean ± SEM. **P < .01; *P < .05. For all Western blotting, we repeated the experiments for three times. We measured grey value of the bands for quantification. Each value was presented as mean ± standard error of the mean (SEM) (Student's *t* test)

To investigate the possible mechanism of action of TLR4, we first studied whether TLR4 influenced histone H3 modification in hLCSCs. We analysed the NF‐kB responsive element (5′‐AGTTGAGGGGACTTTCCCAGGC‐3′) in all the promoters investigated in this study and only found that two NF‐kB‐responsive elements is in CUDR promoter region. As shown in Figure 3A, TLR4 overexpression enhanced the binding of NF‐κB to the non coding RNA CUDR promoter region. As shown in Figure S4, the luciferase activity of CUDR promoter in hLCSCs was higher in TLR4 group than in GFP ctrl group, whereas that was lower in TLR4i group than in RNAi ctrl group. As shown in Figure 3B, compared with GFP ctrl group, TLR4 group enhanced CUDR expression in hLCSCs; however, compared with RNAi ctrl group, TLR4i group decreased that. As shown in Figure 3C,D, compared with GFP ctrl group, TLR4 group enhanced interplay between histone‐lysine N‐methyltransferase (SUV39 h2) and CUDR and interplay between SUV39 h2 and histone H3; however, compared with RNAi ctrl group, TLR4i group decreased those. CUDR knockdown can fully abrogate the action of excessive TLR4 involved in enhancing interplay between SUV39 h2 and histone H3 (Figure 3E). In addition, as shown in Figure 3F, compared with GFP ctrl group, TLR4 group increased formation of trimethyl histone H3 lysine 9 (H3K9me3); however, compared with RNAi ctrl group, TLR4i group decreased that. It should be noticed that neither excessive TLR4 nor TLR4 knockdown can alter the expression of HP1α, HP1β, HP1γ, SUV39 h2 in the hLCSCs; especially, CUDR depletion drastically abolished the action of excessive TLR4 involved in increasing formation of H3K9me3 in hLCSCs (Figure 3G). Compared with GFP ctrl group, TLR4 group increased the interplay between HP1 (HP1α, HP1β and HP1γ) and H3K9me3; however, compared with RNAi ctrl group, TLR4i group decreased that (Figure 3H). Furthermore, as shown in Figure 3I, CUDR depletion can drastically abrogate the TLR4 action involved in increasing interplay between HP1α and H3K9me3. These results suggest TLR4 not only enhances formation of H3K9me3, but also enhances the interplay between HP1 (HP1α, HP1β and HP1γ) and H3K9me3 depending on long non coding RNA CUDR.

### 3.4 TLR4 controls telomere length through H3K9me3

**Figure 4:**
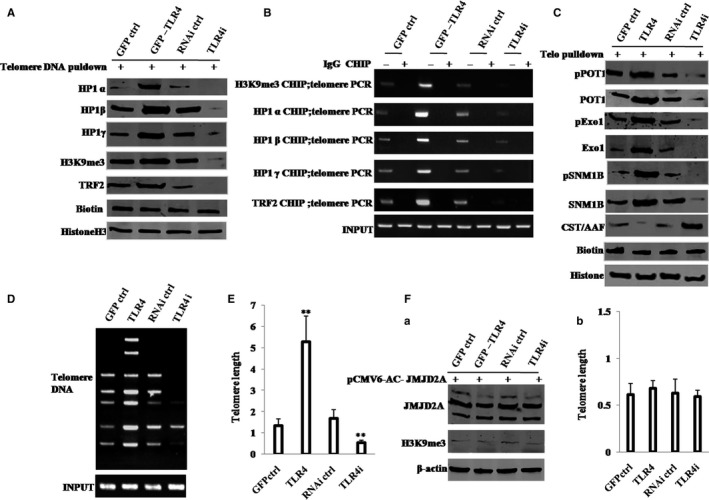
TLR4 controls telomere length through H3K9me3. A, Biotin‐telomere DNA pulldown followed by Western blotting with anti‐HP1α, anti‐HP1β, anti‐HP1γ, anti‐H3K9me3 and anti‐TRF2. Biotin served as INPUT, and histone served as internal control. B, ChIP assay with anti‐HP1α, anti‐HP1β, anti‐HP1γ, anti‐H3K9me3 or anti‐TRF2 followed by PCR with telomere DNA primers. IgG ChIP served as the negative control. C, Biotin‐telomere DNA pulldown followed by Western blotting with anti‐POT1, anti‐pPOT1, anti‐Exo1, anti‐pExo1, anti‐SNM1B, anti‐pSNM1B and anti‐CST/AAF. Biotin as INPUT and histone served as internal control. D, PCR detection of telomere repeat sequence. E, Real‐time PCR detection of telomere length. F, (a) Western blotting with anti‐JMJD2A and anti‐H3K9me3 for four hLCSC lines transfected with pCMV6‐AC‐GFP‐JMJD2A. β‐Actin was used as internal control. (b) Real‐time PCR detection of telomere length for four hLCSC lines transfected with pCMV6‐AC‐GFP‐JMJD2A after inhibition of H3K9me3. Each value was presented as mean ± standard error of the mean (SEM). Bar ± SEM. **P < .01; *P < .05. For all Western blotting, we repeated the experiments for three times. We measured grey value of the bands for quantification. Each value was presented as mean ± standard error of the mean (SEM) (Student's *t* test)

Given that TLR4 increases formation of H3k9me3, we studied whether TLR4 can alter telomere length via H3K9me3. As shown in Figure 4A, compared with GFP ctrl group, TLR4 group increased interplays between the telomere DNA probe and HP1α, HP1β, HP1γ, TRF2, H3K9me3; however, compared with RNAi ctrl group, TLR4i group decreased those. As shown in Figure 4B, compared with GFP ctrl group, TLR4 group increased loadings of HP1α, HP1β, HP1γ, TRF2 and H3K9me3 onto the telomere DNA; however, compared with RNAi ctrl group, TLR4i group decreased those. As shown in Figure 4C, compared with GFP ctrl group, TLR4 group increased interplays between the telomere DNA probe and pTOP1, TOP1, pExo1, Exo1, pSNM1b, NM1b, but decreased interplay between CST/AAF and the telomere DNA probe; however, compared with RNAi ctrl group, TLR4i group decreased interplays between the telomere DNA probe and pTOP1, TOP1, pExo1, Exo1, pSNM1b, SNM1b, but increased interplay between CST/AAF and the telomere DNA probe. As shown in Figure 4D,E, compared with GFP ctrl group, TLR4 group increased telomere length; however, compared with RNAi ctrl group, TLR4i group decreased telomere length. It should be noticed that when H3K9me3 was demethylated by pCMV6‐AC‐GFP‐JMJD2A (a demethylase that demethylates trimethyl histone H3 lysine 9) (Figure 4Fa), the action of TLR4 to alter telomere length was fully abrogated (Figure 4Fb). These results suggest that TLR4 increases telomere length depending on H3K9me3.

### 3.5 TLR4 increases telomerase activity through HP1α‐DNMT3b pathway

**Figure 5:**
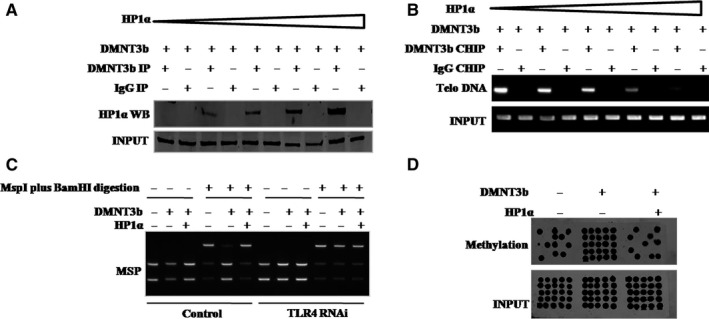
HP1α inhibits DNMT3b activity (A) Co‐IP with anti‐DNMT3b followed by Western blotting with anti‐HP1α in hLCSCs transfected with pcDNA3.1‐DNMT3b. IgG IP served as negative control. Western blotting with DNMT3b served as INPUT. (B) ChIP assay with anti‐DNMT3b followed by PCR with telomere DNA primers in hLCSCs transfected with pcDNA3.1‐DNMT3b and pcDNA3.1‐HP1α. IgG ChIP served as negative control. PCR for telomere DNA served as INPUT. (C) TERRA promoter methylation analysis by MspI plus BamHI digestion in hLCSCs transfected with pcDNA3.1‐DNMT3b and/or pcDNA3.1‐HP1α. (D) TERRA promoter methylation analysis by Methylated DNA Immunoprecipitation (MeDIP)‐Dot blot‐Western blotting with anti‐5‐methylcytosine (5‐mC) in hLCSCs transfected with pcDNA3.1‐DNMT3b or/and pcDNA3.1‐HP1α. Each value was presented as mean ± standard error of the mean (SEM). mean ± SEM. **P < .01; *P < .05. For all Western blotting, we repeated the experiments for three times. We measured grey value of the bands for quantification. Each value was presented as mean ± standard error of the mean (SEM) (Student's *t* test)

To study whether activation of HP1α‐H3K9me3 pathway helps TLR4 alter activity of telomerase involved in DNA methyltransferase (DNMT3b), we first analysed the interrelation between HP1α and DNMT3b in hLCSCs. As shown in Figure 5A, there was an interplay between HP1α and DNMT3b in hLCSCs. Moreover, the formation of HP1α‐DNMT3b complex reduced the interplay between DNMT3b and telomere DNA (lncRNA TERRA promoter) in hLCSCs (Figure 5B). In addition, HP1α inhibited the DNMT3b activity and reduced the methylation on lncRNA TERRA promoter region (Figure 5C,D). However, these actions were fully abrogated when TLR4 was knocked down (Figure 5C). Furthermore, the excessive DNMT3b increased the methylation on lncRNA TERRA promoter region. However, these actions were fully abrogated when TLR4 was knocked down (Figure S5). Moreover, TLR4 knockdown increases the interaction between HP1a and DNMT3b (Figure S6). It suggests that TLR4 knockdown inhibits the activity of DNMT3b through increasing the interaction between HP1a and DNMT3b. Thus, TLR4 knockdown seems to block the activity of DNMT3b by enhancing the interplay between HP1a and DNMT3b.

**Figure 6:**
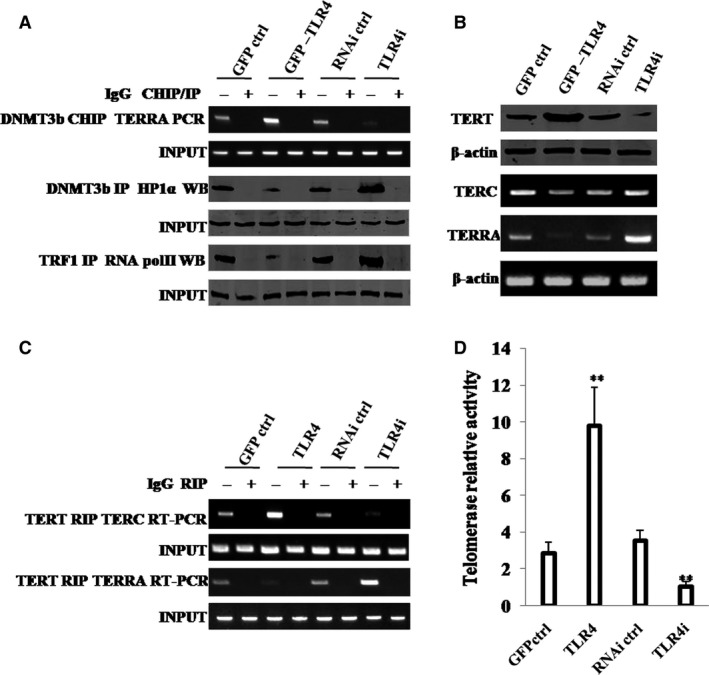
TLR4 increases telomerase activity through HP1α‐DNMT3b. A, (*upper*) ChIP assay with anti‐DNMT3b followed by PCR with TERRA promoter primers. IgG ChIP served as negative control. PCR with TERRA promoter served as INPUT. (*middle*) Co‐IP with anti‐DNMT3b followed by Western blotting with anti‐HP1α. IgG IP served as negative control. Western blotting with anti‐HP1α served as INPUT. *(lower*) Co‐IP with anti‐TRF1 followed by Western blotting with anti‐RNA polII. IgG IP served as negative control. Western blotting with anti‐RNA polII served as INPUT. B, *(upper)* Western blotting with anti‐TERT. (*lower*) RT‐PCR with TERC and TERRA primers for four hLCSC lines. β‐Actin served as internal control. C, RIP with anti‐TERT followed by RT‐PCR with TERC and TERRA primers. IgG RIP served as negative control. RT‐PCR for TERC or TERRA served as INPUT. D, Telomerase assay with TRAP method. Each value was presented as mean ± standard error of the mean (SEM). Bar ± SEM. **P < .01; *P < .05. For all Western blotting, we repeated the experiments for three times. We measured grey value of the bands for quantification. Each value was presented as mean ± standard error of the mean (SEM) (Student's *t* test)

Given that HP1α inhibited DNMT3b activity and reduced methylation on TERRA promoter region, which is associated with TLR4, we studied whether TLR4 could alter telomerase activity. As shown in Figure 6A, compared with GFP ctrl group, TLR4 group increased loading of DNMT3b on the TERRA promoter region, but decreased interplay between DNMT3b and HP1α, and interplay between TRF1 and RNA PolII; however, compared with RNAi ctrl group, TLR4i group decreased loading of DNMT3b on the TERRA promoter region, but increased interplay between DNMT3b and HP1α, and interplay between TRF1 and RNA PolII. As shown in Figure 6B, compared with GFP ctrl group, TLR4 group increased expression of TERT, but decreased expression of TERRA; however, compared with RNAi ctrl group, TLR4i group decreased expression of TERT, but increased expression of TERRA. It is worth noting that neither TLR4 overexpression nor TLR4 knockdown can alter the expression of TERC (Figure 6B). Moreover, compared with GFP ctrl group, TLR4 group increased interplay between TERT and TERC, but decreased interplay between TERT and TERRA; however, compared with RNAi ctrl group, TLR4i group decreased interplay between TERT and TERC, but increased interplay between TERT and TERRA (Figure 6C). Finally, compared with GFP ctrl group, TLR4 group increased telomerase activity in hLCSCs; however, compared with RNAi ctrl group, TLR4i group decreased that (Figure 6D). These results suggest that TLR4 increases telomerase activity via HP1α‐DNMT3b pathway.

### 3.6 HP1 isoforms (HP1α, HP1β and HP1γ) are required for TLR4 oncogenic action

Given that TLR4 enhances the interplay between HP1 isoforms (HP1α, HP1β and HP1γ), increases the telomere length by HP1‐H3K9me3 and increases telomerase activity by HP1‐DNMT3b pathway, we studied whether HP1 (HP1α, HP1β and HP1γ) could determine the TLR4 oncogenic function. To analyse the formation of tri‐complex of HP1α‐HP1β‐HP1γ, we performed the repeat co‐immunoprecipitation (IP) experiments, that is, first, HP1α IP and second, HP1β repeat IP with the immunoprecipitates from HP1α IP.

**Figure 7:**
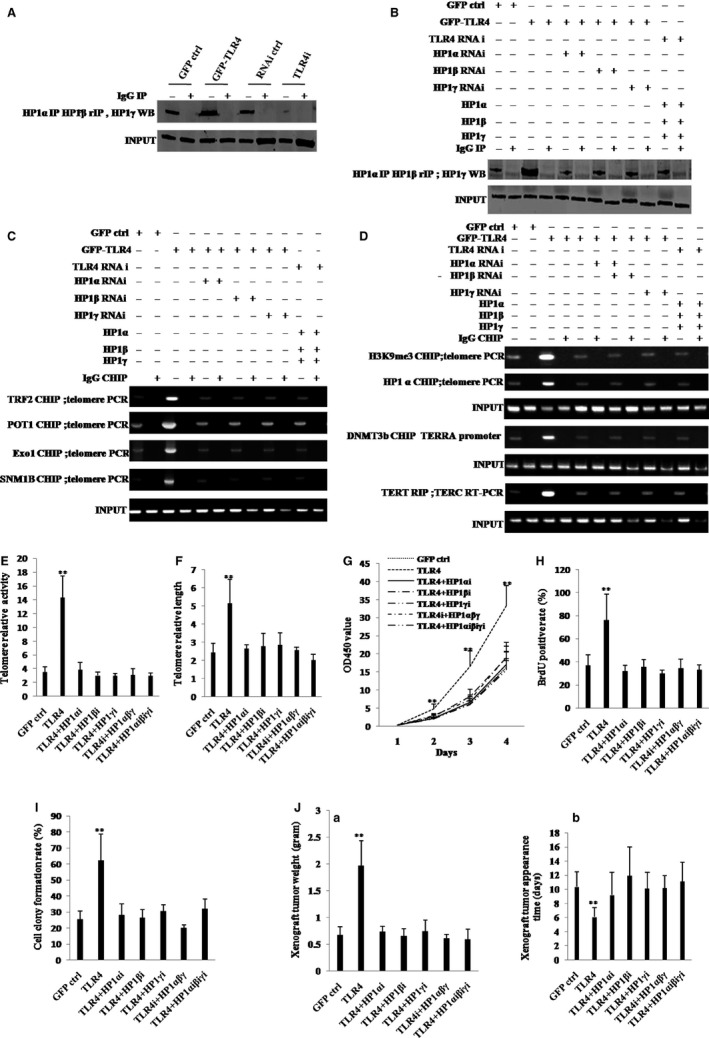
HP1 isoforms are required for TLR4 oncogenic action. A Repeat Co‐IP with anti‐HP1α or anti‐HP1β followed by Western blotting with anti‐HP1γ. IgG IP served as negative control. Western blotting with anti‐HP1γ served as INPUT. B, Repeat Co‐IP with anti‐HP1α or anti‐HP1β followed by Western blotting with anti‐HP1γ. IgG IP served as negative control. Western blotting with anti‐HP1γ served as INPUT. C, ChIP assay with anti‐TRF2, anti‐POT1, anti‐Exo1, anti‐SNM1B followed by PCR with telomere promoter primers. IgG ChIP served as negative control. PCR with telomere promoter primers served as INPUT. D, (*upper*) ChIP assay with anti‐H3K9me3, anti‐HP1α, anti‐DNMT3b followed by PCR with telomere or TERRA promoter primers. IgG ChIP served as negative control. PCR with telomere or TERRA promoter primers served as INPUT. (*lower*) RIP with anti‐TERT followed by RT‐PCR with TERC primers. IgG RIP served as negative control. RT‐PCR for TERC served as INPUT. B‐D, hLCSC lines: GFP ctrl group, TLR4 group, TLR4i group, and hLCSC lines whose HP1α, HP1β and HP1γ are overexpressed or depleted. The even number lanes are results from IgG controls. E, Telomerase activity assay with TRAP method primers. F, Real‐time PCR detection of telomere length. G, Cell growth assay using CCK8. H, Cell BrdU staining assay. I, Soft‐agar colony formation assay. J, (a) The wet weight of xenografted tumours from mouse. (b) PCNA staining (DAB staining, original magnification × 100). E‐J, hLCSC lines: GFP ctrl group, TLR4 group, TLR4 group transfected with pGFP‐V‐RS‐HP1α, pGFP‐V‐RS‐HP1β, pGFP‐V‐RS‐HP1γ and pGFP‐V‐RS‐HP1(α, β, γ), and TLR4i group transfected with pcDNA‐(HP1α, HP1β and HP1γ). Each value was presented as mean ± standard error of the mean (SEM). Mean ± SEM. **P < .01; *P < .05. For all Western blotting, we repeated the experiments for three times. We measured grey value of the bands for quantification. Each value was presented as mean ± standard error of the mean (SEM) (Student's *t* test)

As shown in Figure 7A,B, TLR4 overexpression increased interaction among HP1α, HP1β and HP1γ in hLCSCs, whereas TLR4 knockdown decreased that. As shown in Figure S7, we show the specificity of each knockdown reagent (HP1α, HP1β and HP1γ). The expression of HP1α was significantly reduced only in HP1α knockdown group, the expression of HP1β was significantly reduced only in HP1β knockdown group, and the expression of HP1γ was significantly reduced only in HP1γ knockdown group. Strikingly, TLR4 overexpression increased loadings of TRF2, POT1, Exo1 and SNM1b on telomere DNA in hLCSCs, whereas TLR4 knockdown decreased those (Figure 7C). TLR4 overexpression increased loading of H3K9me3 and HP1α on telomere DNA, loading of DNMT3b on TERRA promoter region and interaction between TERT and TERC in hLCSCs, whereas TLR4 knockdown decreased those (Figure 7D). Furthermore, TLR4 overexpression led to increase in telomerase activity and telomere length; however, these actions were fully abrogated by depletion of HP1α, HP1β, HP1γ, HP1(α, β, γ) (Figure 7E and F). Moreover, overexpression of HP1α, HP1β, HP1γ did not significantly alter the telomerase activity and telomere length in hLCSCs whose TLR4 was depleted (Figure 7E,F, TLR4i+HP1αβγ). Furthermore, excessive TLR4 significantly increased the level of phosphorylation of HP1α, HP1β, HP1γ. However, when protein phosphatase PP1 was expressed in the TLR4 overexpressing liver cancer stem cells, excessive TLR4 could significantly not alter the level of phosphorylation of HP1α, HP1β, HP1γ (Figure S8A). Moreover, excessive TLR4 significantly increased the length of telomere (2.427 ± 0.732 vs 7.343 ± 0.512, *P* = .0088 < .01). However, when protein phosphatase PP1 was expressed in the TLR4 overexpressing liver cancer stem cells, excessive TLR4 could significantly not alter the length of telomere (2.427 ± 0.732 vs 2.123 ± 0.26, *P* = .193 > .05) (Figure S8B). And excessive TLR4 significantly increased the activity of telomerase (6.25 ± 1.37 vs 25.08 ± 3.82, *P* = .006051 < .01). However, when protein phosphatase PP1 was expressed in the TLR4 overexpressing liver cancer stem cells, excessive TLR4 could significantly not alter the activity of telomerase (6.25 ± 1.37 vs 5.71 ± 1.91, *P* = .134 > 0.05) (Figure S8C). TLR4 overexpression promoted cell proliferation, proportion of BrdU‐positive cells, colony formation ability and xenograft tumour formation ability (including xenograft weight and tumour appearance time) of hLCSCs; however, these actions were fully abrogated by depletion of HP1α, HP1β, HP1γ, HP1(α, β, γ)(Figure 7G‐J). Furthermore, overexpression of HP1α, HP1β, HP1γ did not significantly alter cell proliferation, proportion of BrdU‐positive cells, colony formation ability and xenograft tumour formation ability of hLCSCs whose TLR4 was knocked down (Figure 7G‐J, TLR4i+HP1αβγ). These results suggest that TLR4‐dependent characteristics of hLCSCs are abrogated when HP1 isoforms (α, β and γ) are depleted, and tri‐complexes of HP1 isoforms (α, β and γ) are required for TLR4 oncogenic action.

## 4 DISCUSSION

**Figure 8:**
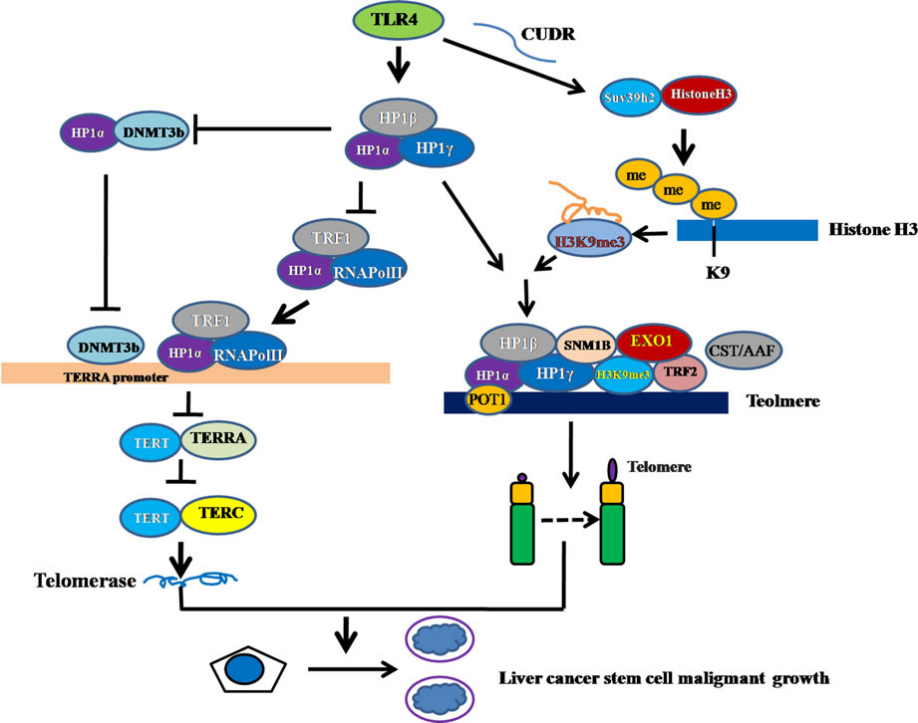
Schematic illustration of the role of TLR4 in malignant proliferation and growth of hLCSCs in vitro and in vivo. Mechanistically, TLR4 promotes expression of SUV39 h2 (which methylates H3K9 to form H3K9me3) and then increases formation of H3K9me3‐HP1‐TRF2 complex at the telomeric locus under mediation by long non coding CUDR. At the telomeric locus, this complex promotes binding of POT1, pPOT1, Exo1, pExo1, SNM1B and pSNM1B but prevents binding of CST/AAF to telomere, thus controlling telomere and maintaining telomere length. Furthermore, TLR4 inhibits the interaction between HP1α and DNMT3b, which limits RNA polymerase II deposition on TERRA promoter region and its elongation, thus inhibiting transcription of TERRA. Ultimately, TLR4 enhances the telomerase activity by reducing interplay between TERT and TERRA but enhancing the interplay between TERT and TERC

Cancer stem cells (CSCs) are involved in tumour initiation, progression, recurrence and metastasis. TLR4, an inflammatory factor receptor, has been reported to play a significant role in various cancers. However, the regulatory mechanism of TLR4 has not been elucidated. To our knowledge, this study might be the first to demonstrate that TLR4 controls telomeres through HP1 isoforms in hLCSCs. As shown in Figure 8, we provide evidence that TLR4 promotes the malignant proliferation and growth of hLCSCs in vitro and in vivo. Strikingly, our results also reveal that tri‐complexes of HP1 isoforms (α, β and γ) are required for TLR4 oncogenic action. The detailed discussion is as follows.

First, accumulating evidence indicates that TLR4 might stimulate carcinoma initiation and progression.^27^, ^28^ Our present results are consistent with these reports and provide novel evidence for an active role of TLR4 in promoting liver cancer stem cell growth. This evidence is based on results from two parallel sets of experiments: (*i*) TLR4 facilitates proliferation of hLCSCs in vitro, and (*ii*) TLR4 accelerates growth of hLCSCs in vivo.

In addition, epigenetic engineering shows that a human centromere can resist silencing, which is mediated by H3K27me3/K9me3.^29^ Suppressor of variegation 3‐9 homologue 2 (SUV39 h2) is a SET domain‐containing histone methyltransferase that is up‐regulated in solid cancers,^30^ which inhibits polyubiquitination in human cancer cells by methylating and stabilizing LSD1.^31^ Our results suggest that TLR4 promotes trimethylation of histone H3 at lysine 9. This is based on several results of TLR4 overexpression or knockdown in hLCSCs: (*i*) TLR4 enhances interplay between long non coding RNA CUDR and SUV39 h2, and (*ii*) TLR4 drives more SUV39 h2 to H3K9 site, producing more H3K9me3. Heterochromatin causes epigenetic repression that can be transmitted through multiple cell divisions.

Another significant finding is that TLR4 increases telomere length. This is based on several results of TLR4 overexpression or knockdown in hLCSCs: (*i*) TLR4 enhances interplay between long non coding RNA CUDR and SUV39 h2, (*ii*) TLR4 promotes the formation of tri‐complexes including H3K9me3, HP1α and TRF2 on the telomere, and (*iii*) TLR4 promotes binding of POT1, pPOT1, Exo1, pExo1, SNM1B and pSNM1B but prevents binding of CST/AAF to telomere. Several studies indicate that the structures of the SNM1A and SNM1B/Apollo nuclease domains reveal a potential basis for their distinct DNA processing activities.^32^ Exo1 extensively resects both ends of telomere, generating transient long 3′ overhangs in S/G2.^20^ Furthermore, TERRA promotes telomere shortening through exonuclease 1‐mediated resection of chromosome ends.^33^

Furthermore, accumulating evidence suggests that the human TERT contributes to cell physiology independently of its ability to elongate telomeres,^34^ and the shelterin protein TRF2 is essential for chromosome‐end protection.^35^ Our previous findings suggest that SET1A plus CUDR increased TRF2 expression at the transcriptional and translational level and its activity through H3K4me3.^36^ Particularly, TERRA RNA interacts with several telomere‐associated proteins, including TRF1 and TRF2.^37^ Our results also show that TLR4 enhances telomerase activity. This is based on several results of hLCSCs: (*i*) HP1α inhibits activity of DNMT3b that alters methylation of TERRA promoter by forming the HP1α‐DNMT3b complexes, (*ii*) TLR4 inhibits TERRA expression by decreasing the HP1α‐DNMT3b complexes, and (*iii*) TLR4 increases telomerase activity through increasing interaction between TERT and TREC but decreasing the interaction between TERT and TERRA.

Also, some studies show that HP1 mediates the recognition and destruction of heterochromatic RNA transcripts^38^ and promotes tumour suppressor BRCA1 functions during the DNA damage response.^39^ Strikingly, heterochromatic‐silencing factors preclude histone turnover to promote silencing and inheritance of repressive chromatin.^40^ Particularly, HP1α nucleates with high affinity independently of H3K9me in promoters of active genes and then spreads via H3K9 methylation and transient looping contacts with those H3K9me target sites.^41^ Our findings suggest tri‐complexes of HP 1 isoforms (α, β and γ) are required for TLR4 oncogenic action. This is based on several results of hLCSCs: (*i*) TLR4 overexpression leads to increase in interaction among HP1α, HP1β and HP1γ in hLCSCs, whereas TLR4 knockdown leads to decrease in that, (*ii*) TLR4 overexpression leads to increase in loading of TRF2, POT1, Exo1 and SNM1b on the telomere in hLCSCs, whereas TLR4 knockdown leads to decrease in that. However, this action can be fully abrogated by depletion of HP1α, HP1β and HP1γ, (*iii*) TLR4 overexpression leads to increase in loading of H3K9me3 and HP1α on the telomere DNA, loading of DNMT3b on the TERRA promoter region and interaction between TERT and TERC in hLCSCs, whereas TLR4 knockdown leads to decrease in those. However, this action can be fully abrogated by depletion of HP1α, HP1β and HP1γ, (*iv*) TLR4 overexpression leads to increase in telomerase activity and telomere length. However, this action can be fully abrogated by depletion of HP1α, HP1β and HP1γ, and (*v*) TLR4 overexpression leads to increase in cell proliferation, colony formation and xenograft tumour formation ability. However, this action can be fully abrogated by depletion of HP1α, HP1β and HP1γ.

Finally, the function of TLR4 in liver cancer stem cells should be further explored. We postulate that TLR4 functions may be independent of NF‐κB, which is a key transcriptional regulator involved in inflammation, cell proliferation, survival and transformation. In this respect, outstanding questions include the following: (*i*) What is the mechanism of oncogenic action of TLR4? (*ii*) How does TLR4 cooperate with HP1? and (*iii*) Does TLR4 regulate a series of molecular events during the malignant growth of liver cancer stem cells? Answering these questions will help understand the mechanism underlying the malignant differentiation of liver stem cells. In summary, our data indicate that TLR4 promotes liver cancer stem cells malignant progression by altering telomere length. These results provide insight into a novel link between TLR4 and hepatocarcinogenesis and also have diagnostic and prognostic implications.

## CONFLICT OF INTEREST

The authors declare no conflict of interests.
