# Supplementary figures and images for: Inflammatory factor receptor Toll‐like receptor 4 controls telomeres through heterochromatin protein 1 isoforms in liver cancer stem cell
Source: J Cell Mol Med. 2018 Mar 30;22(6):3246–58. doi: 10.1111/jcmm.13606 (PMC5980149; doi:10.1111/jcmm.13606)

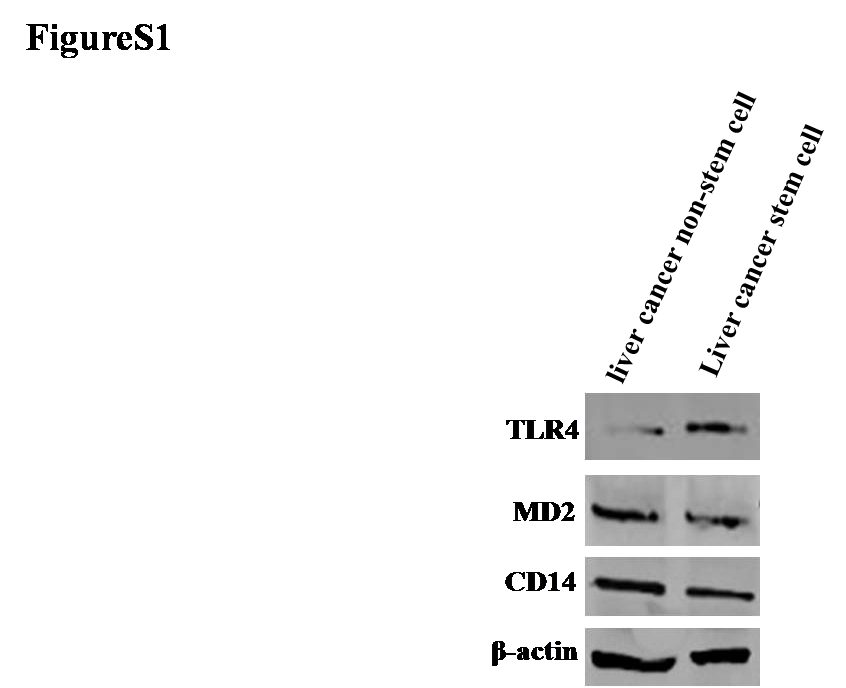

Supplement: Supplementary file 1 [file JCMM-22-3246-s001.tif]

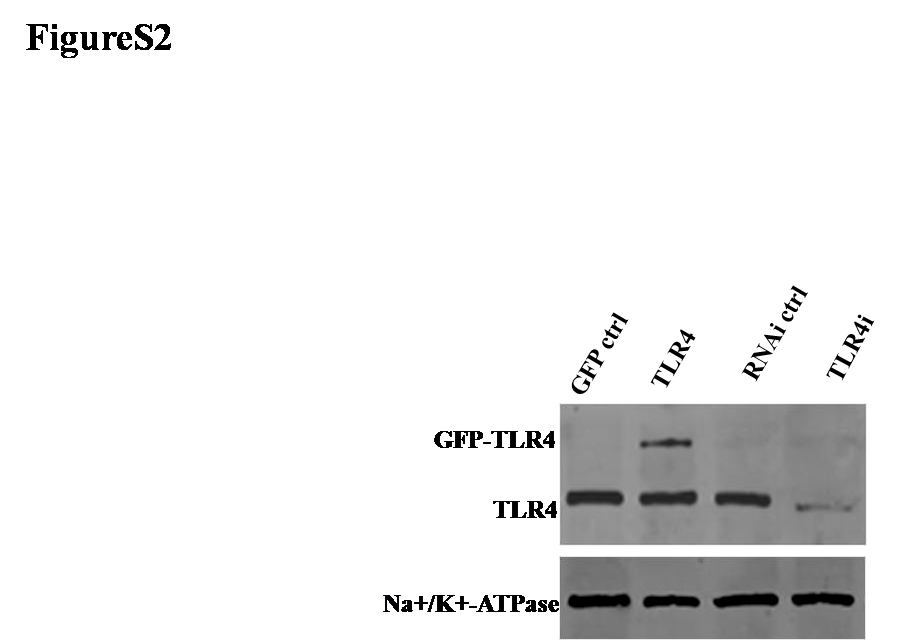

Supplement: Supplementary file 2 [file JCMM-22-3246-s002.tif]

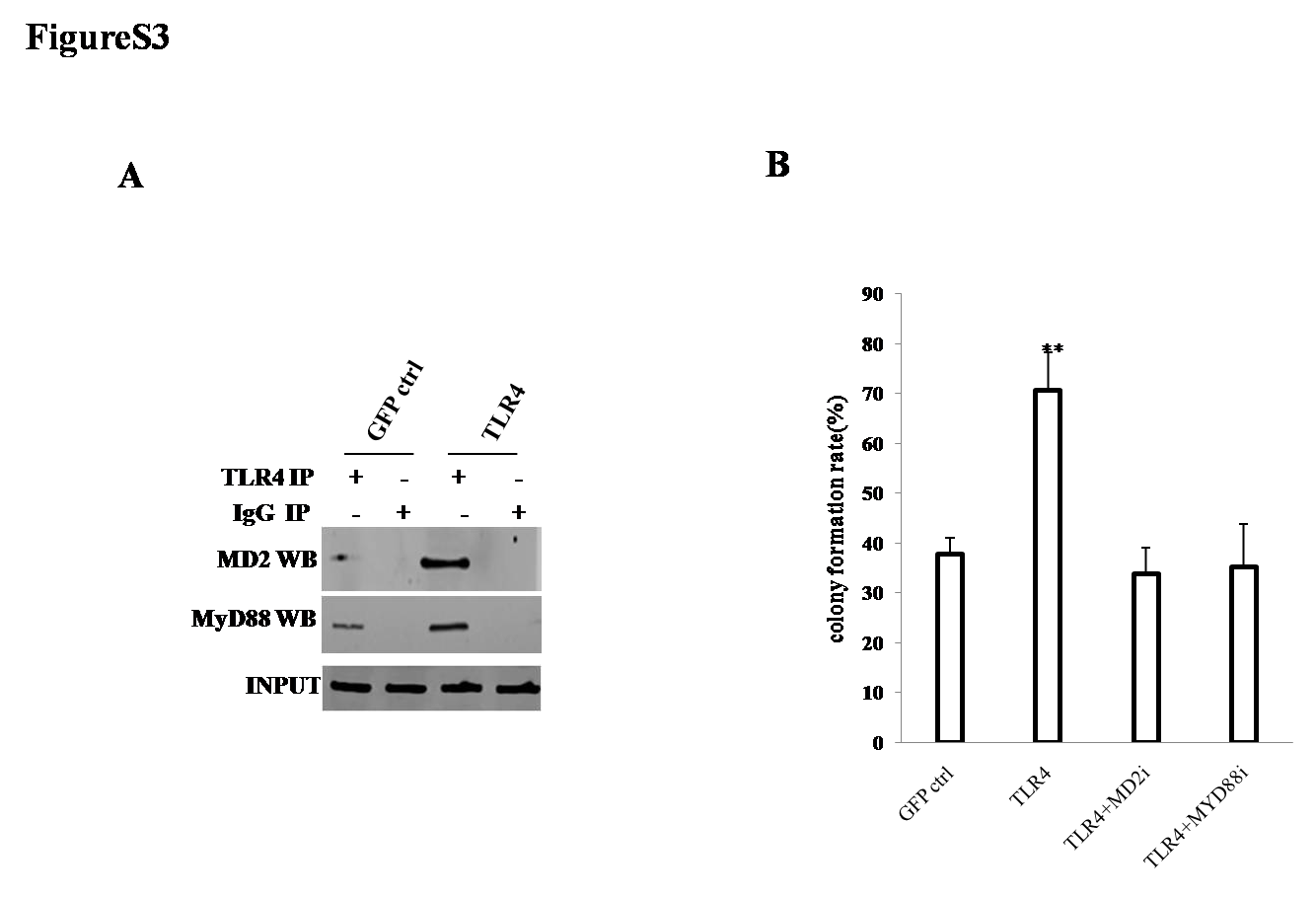

Supplement: Supplementary file 3 [file JCMM-22-3246-s003.tif]

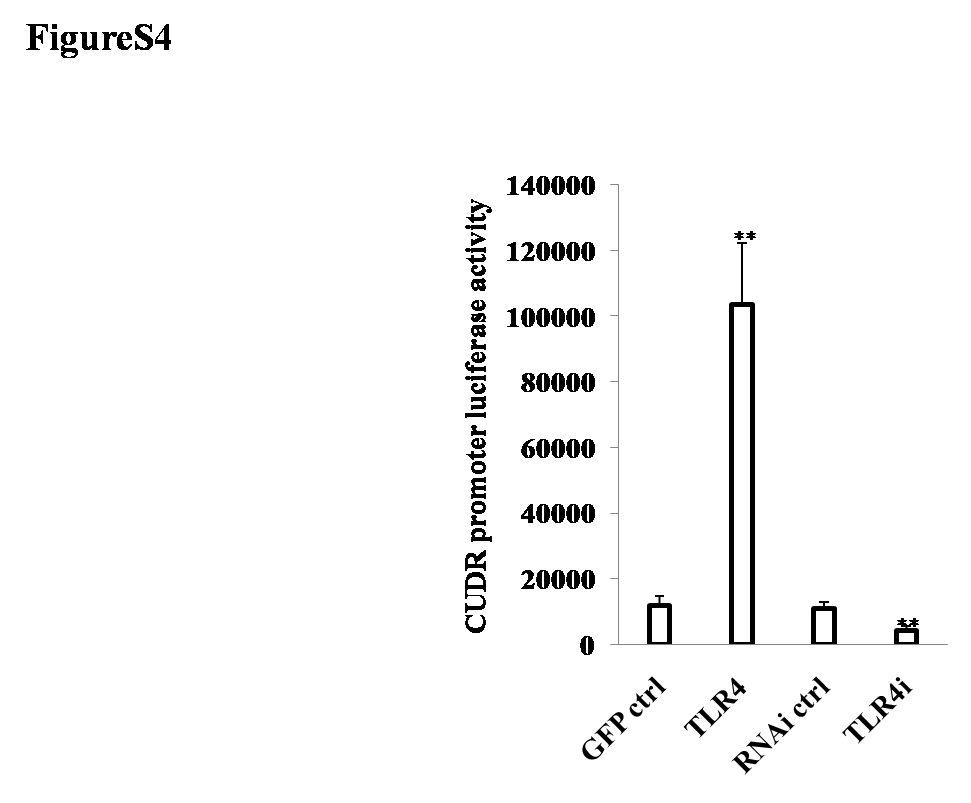

Supplement: Supplementary file 4 [file JCMM-22-3246-s004.tif]

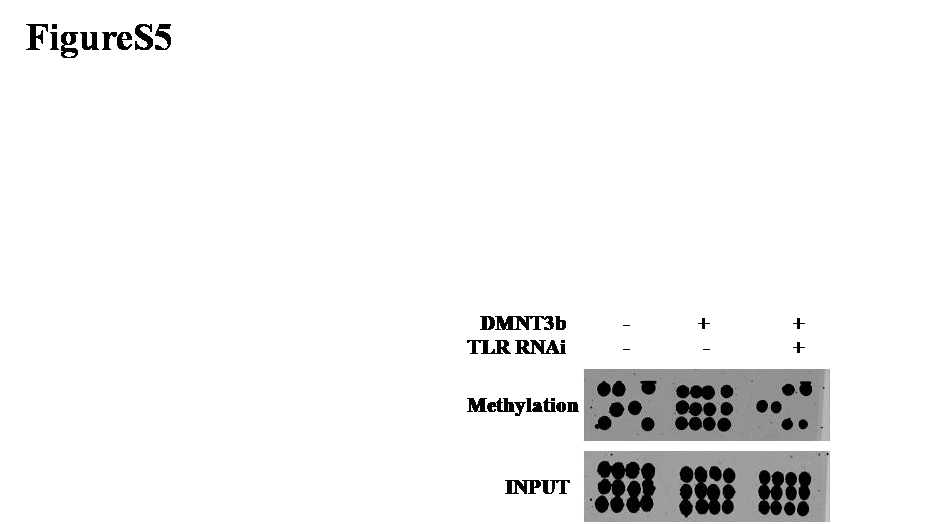

Supplement: Supplementary file 5 [file JCMM-22-3246-s005.tif]

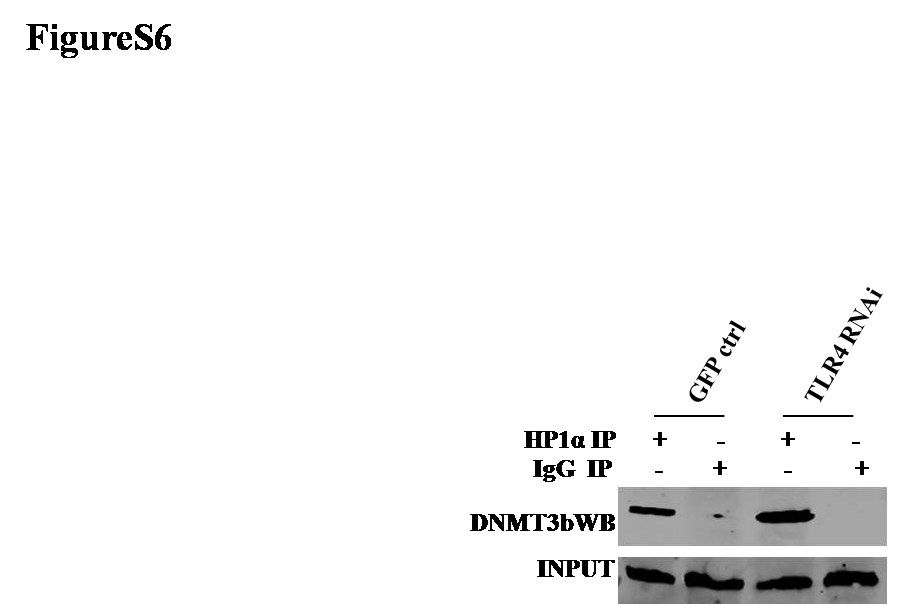

Supplement: Supplementary file 6 [file JCMM-22-3246-s006.tif]

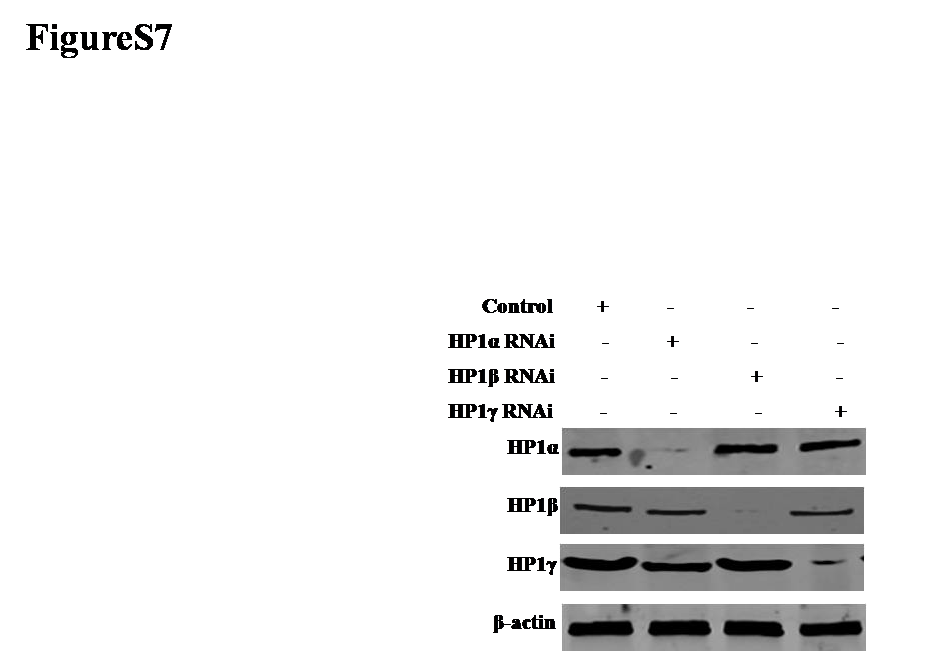

Supplement: Supplementary file 7 [file JCMM-22-3246-s007.tif]

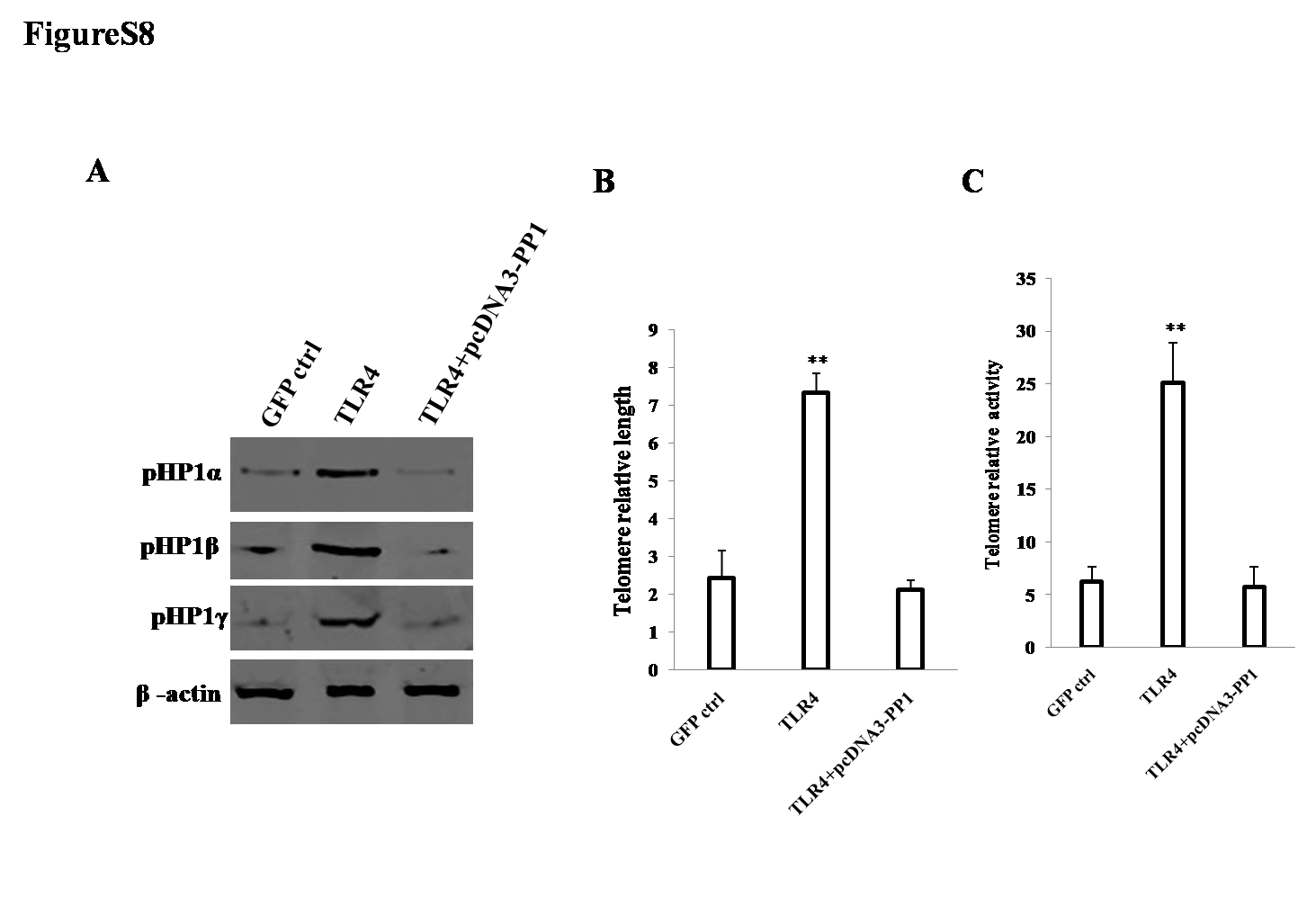

Supplement: Supplementary file 8 [file JCMM-22-3246-s008.tif]
