# Supplementary material for: Inflammatory factor receptor Toll‐like receptor 4 controls telomeres through heterochromatin protein 1 isoforms in liver cancer stem cell
Source: J Cell Mol Med. 2018 Mar 30;22(6):3246–58. doi: 10.1111/jcmm.13606 (PMC5980149; doi:10.1111/jcmm.13606)
Supplement: Supplementary file 9 [file JCMM-22-3246-s009.docx]

**SUPPLEMENTAL FIGURE LEGENDS**

**FigureS1** Western blotting analysis of expression of MD2, CD14 in hLCSCs and non-hLCSCs. β-actin as internal control.

**FigureS2** Western blotting analysis of cell surface expression of TLR4 in four hLCSC lines(GFP ctrl,TLR4,RNAi ctrl ,TLR4i). Na+/K+-ATPase as internal control.

**FigureS3 A.**co-immunoprecipitation(co-IP) with anti-TLR4 followed by western blotting with anti-MD2,anti-MyD88.IgG IP served as the negative control. Western blotting with anti-TLR2 served as INPUT. **B.** Soft-agar colony formation assay in four hLCSC lines(GFP ctrl,TLR4,TLR4+MD2i,TLR4+MyD88).

**FigureS4** CUDR promoter luciferase activity assay in four hLCSC lines. (GFP ctrl,TLR4,RNAi ctrl ,TLR4i). Each value was presented as mean ± standard error of the mean (SEM). Bar ± SEM. **, P<0.01; *, P<0.05.

**FigureS5** TERRA promoter methylation analysis by Methylated DNA Immunoprecipitation (MeDIP)-Dot blot-Western blotting with anti-5-Methylcytosine (5-mC) in hLCSCs transfected with pcDNA3.1-DNMT3b or/and pGFP-V-RS-TLR4.

**FigureS6** co-immunoprecipitation(co-IP) with anti-HP1α followed by western blotting with anti-DNMT3b in two hLCSC lines(RNAi ctrl, pGFP-V-RS-TLR4).IgG IP served as the negative control. Western blotting with anti- HP1α served as INPUT.

**FigureS7** Western blotting analysis of expression of HP1α, HP1β,HP1γ in hLCSCs(RNAi ctrl, pGFP-V-RS- HP1α, pGFP-V-RS-HP1β, pGFP-V-RS-HP1γ). β-actin as internal control.

**FigureS8 A.**Western blotting analysis of expression of pHP1α, pHP1β,pHP1γ in hLCSCs(GFP ctrl, pCMV6-GFP-TLP4, pCMV6-GFP-TLR4 plus pcDNA3-PP1). β-actin as internal control. **B.** Real-time PCR detection of telomere length. **C.**Telomerase activity assay with TRAP method primers. Each value was presented as mean ± standard error of the mean (SEM). Bar ± SEM. **, P<0.01; *, P<0.05.
